# Contribution of p62/SQSTM1 to PDGF-BB-induced myofibroblast-like phenotypic transition in vascular smooth muscle cells lacking *Smpd1* gene

**DOI:** 10.1038/s41419-018-1197-2

**Published:** 2018-11-19

**Authors:** Peng Zhang, Yinglu Guan, Jiajie Chen, Xiang Li, Bradley K. McConnell, Wei Zhou, Krishna M. Boini, Yang Zhang

**Affiliations:** 1 Department of Oncology, Tongji Hospital, Tongji Medical College, Huazhong University of Science and Technology, Wuhan, China; 2 Department of Pharmacological and Pharmaceutical Sciences, College of Pharmacy, University of Houston, Houston, USA

## Abstract

Accumulating evidence indicates a critical role of autophagy in regulating vascular smooth muscle cell (SMC) homeostasis in atherogenesis. However, little is known about the modulatory role of autophagy in PDGF-BB-induced SMC transition towards the synthetic phenotype and extracellular matrix remodeling. We recently demonstrated that acid sphingomyelinase (ASM, encoded by *Smpd1* gene) controls autophagy maturation in coronary arterial SMCs. Here, we demonstrate that PDGF-BB stimulation causes a myofibroblast-like non-canonical synthetic phenotype transition in *Smpd1*^*−/−*^ SMCs. These non-canonical phenotypic changes induced by PDGF-BB in *Smpd1*^*−/−*^ SMCs were characterized by increased expression of fibroblast-specific protein (FSP-1), massive deposition of collagen type I, decreased cell size, elevated inflammatory status with enhanced cytokine release and adhesion molecule expression. Mechanistically, PDGF-BB induces prolonged Akt activation that causes decreased autophagosome biogenesis and thereby exaggerates p62/SQSTM1 accumulation in *Smpd1*^*−/−*^ SMCs. More importantly, Akt inhibition or p62/SQSTM1 gene silencing attenuates PDGF-BB-induced phenotypic changes in *Smpd1*^*−/−*^ SMCs. This first demonstration of a p62/SQSTM1-dependent myofibroblast-like phenotypic transition in *Smpd1*^*−/−*^ SMCs suggests that ASM-mediated autophagy pathway contributes to maintaining the arterial smooth muscle homeostasis in situation of vascular remodeling during atherosclerosis.

## Introduction

Vascular smooth muscle cell (SMC) is a highly specialized and differentiated cell and major constitute of blood vessels. SMCs within the adult blood vessel possess contractile phenotype and exhibit a very low synthetic activity. SMCs can switch from a differentiated (contractile) phenotype to a dedifferentiated (synthetic) state that SMCs become proliferative and migratory. The deregulation of SMC phenotypic plasticity is a pathogenic basis for vascular diseases such as atherosclerosis^1^. In addition to “canonical” synthetic phenotype, SMCs can also switch to other non-canonical phenotypes such as myofibroblast-like phenotypes^2^. Modulation of SMCs toward a myofibroblastic phenotype can occur within the human plaque^3^. It has been proposed that myofibroblastic transition contributes to the formation of atheromatous plaque, a complex inflammatory and fibroproliferative process^4^. Myofibroblasts are cells normally found in pathological situation that are responsible for the production extracellular matrix components (type I and III collagens and fibronectin) as well as inflammatory cytokines at the site of fibrosis^5,6^. Myofibroblasts have acquired a phenotype intermediate between fibroblasts and SMCs. They are contractile cells expressing α-smooth muscle actin (α-SMA), the actin isoform typical of vascular SMCs, and have a flattened and irregular morphology. In general, myofibroblasts originate from local fibroblasts, however, they are also derived from local SMCs in certain pathological settings. So far, the signaling pathways and mediators through which the SMCs switch to the inflammatory myofibroblasts remain largely undefined.

Acid sphingomyelinase (ASM), encoded by *Smpd1* gene, is a lysosome hydrolase that metabolizes sphingomyelin to ceramide and phosphorylcholine^7^. Clinical studies reported that the Niemann-Pick disease patients with deficient ASM activity had high incidences of coronary atherosclerosis^8,9^, suggesting that ASM activity is crucial for preventing atherogenesis in humans. Consistently, adenovirus-mediated ASM expression decreased the lesion formation in atherosclerotic ApoE^-/-^ mice^10^. In macrophages, ASM-mediated sphingomyelin hydrolysis prevents the retention of cholesterol in lysosomes and foam cell formation^11,12^. Conversely, ASM promotes aggregation and uptake of lipoproteins by arterial-wall macrophages leading foam cell formation^13,14^. It seems ASM participates in various stages of foam cell formation with either anti- or pro-atherogenic roles. Autophagy is a nonstop, reparative, and life-sustaining way to maintain normal cellular homeostasis^15^. Our recent studies demonstrate that ASM is needed for lysosome trafficking and autophagy maturation in vascular SMCs treated with atherogenic oxidized cholesterol^16,17^. ASM exerts its anti-atherogenic effect via modulating autophagy that induces SMCs to a more differentiated contractile phenotype, thereby decreasing cell proliferation and preventing fibrosis^18^. It is intriguing to explore the precise role of ASM and autophagy signaling in modulating myofibroblastic transition in SMCs.

Platelet-derived growth factor-BB (PDGF-BB) is a potent inducer of SMC phenotype switching. The expression of PDGF-BB in vasculature is upregulated in situation of vascular remodeling during atherosclerosis. The present study first identified the role of ASM in SMC myofibroblastic transition by PDGF-BB and then characterized its mechanism of action. As such, we investigated the effects of ASM deficiency by genetic ablation of *Smpd1* gene on SMC proliferation, migration, morphological change, extracellular matrix secretion, and inflammatory cytokine production in response to PDGF-BB with in vitro and ex vivo analyses. Moreover, we used recombinant lentiviral vector targeting p62/SQSTM1, a specific autophagy substrate, to test the role of the ASM-autophagy-p62/SQSTM1 axis in myofibroblastic transition by PDGF-BB.

## Results

### Effects of *Smpd1* gene ablation on PDGF-BB-induced phenotypic modulation of SMCs

**Fig. 1:**
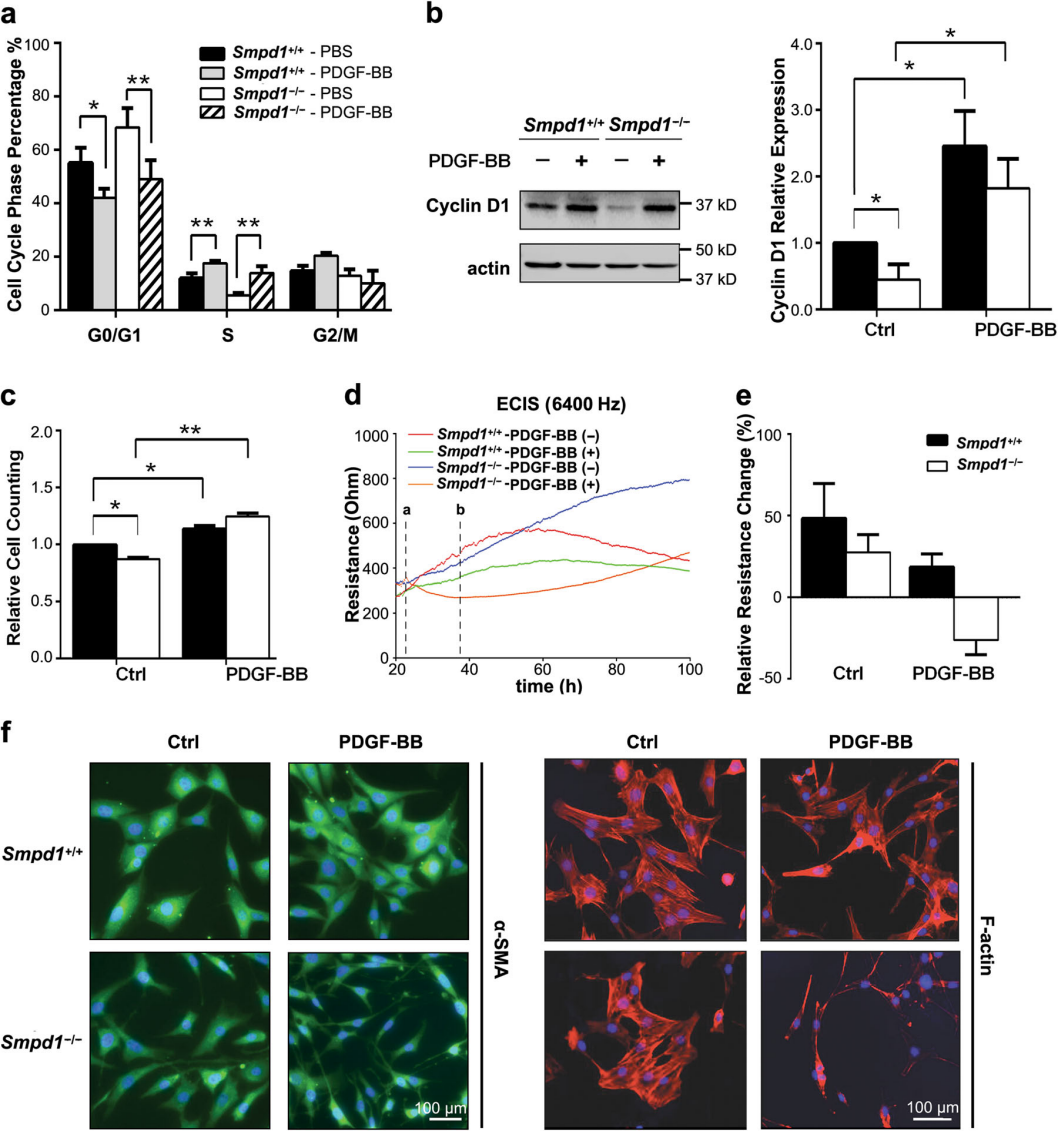
PDGF-induced changes in proliferation and morphology in *Smpd1*^*+/+*^ and *Smpd1*^*−/−*^ SMCs. Vascular SMCs were treated with PDGF-BB (30 ng/ml) for 24 h or indicated time period. **a** Cell cycle analysis of *Smpd1*^*+/+*^ and *Smpd1*^*−/−*^ SMCs in the presence or absence of PDGF-BB (30 ng/ml) by flow cytometry. The bar graph shows the percentage of *Smpd1*^*+/+*^ and *Smpd1*^*−/−*^ nuclei in the G0/1, S, and G2/M phase of the cell cycle. ^*^P < 0.05, ^**^P < 0.01 (*n* = 4, Two-way ANOVA)**. b** Immunoblotting analysis and summarized data show the expression of cyclin D1 in *Smpd1*^*+/+*^ and *Smpd1*^*−/−*^ SMCs in the presence or absence of PDGF-BB. ^*^P < 0.05 (*n* = 4, Two-way ANOVA). **c** Flow cytometry-based cell counting of *Smpd1*^*+/+*^ and *Smpd1*^*−/−*^ SMCs treated with or with PDGF-BB. ^*^P < 0.05, ^**^P < 0.01 (*n* = 4, Two-way ANOVA). **d**, **e** ECIS analysis of cell morphology. Cells were seeded in ECIS array chamber for 24 h before addition of PDGF-BB (dotted line a). The impedance of *Smpd1*^*+/+*^ and *Smpd1*^*−/−*^ SMCs was monitored by ECIS assay within 100 h. The dotted line b indicates the lowest impedance value of the curve for *Smpd1*^*−/−*^ SMCs treated with PDGF-BB. Summarized data in **e** show the resistance changes of between dotted line a and b (*n *= 4). **f** Represent immunofluorescence images for α-SMA (green) or F-actin (red) with DAPI (blue) in SMCs. The experiment was repeated at least three times with similar results

PDGF-BB is a potent inducer of phenotypic transition of SMCs towards a synthetic phenotype by modulating cell cycle regulators including cyclin D1^19,20^. In *Smpd1*^+/+^ SMCs, PDGF-BB (30 ng/ml) significantly decreased percentage of cells in G0/G1 phase and increased percentage of cells in S phase (Fig. 1a), and increased cyclin D1 (Fig. 1b) and cell numbers (Fig. 1c). These results confirmed that PDGF-BB promoted cell cycle progression and proliferation in *Smpd1*^+/+^ SMCs. *Smpd1*^*−/−*^ SMCs exhibited lower level of cyclin D1 and less number of cells in S phase at the basal condition. However, PDGF-induced changes in cell cycle, cyclin D1 expression, or cell numbers by PDGF-BB were similar in *Smpd1*^*−/−*^ and *Smpd1*^+/+^ SMCs. These data suggest that *Smpd1* gene deletion may affect SMC proliferation at the basal level but is insufficient to inhibit PDGF-BB-induced proliferation.

Next, the effect of *Smpd1* gene deficiency on SMC morphology was examined using ECIS technology. As shown in Fig. 1d, the impedance of untreated *Smpd1*^+/+^ SMCs time-dependently increased to a plateau where cell confluence was achieved. Compared to untreated control, PDGF-BB moderately attenuated the impedance in *Smpd1*^+/+^ SMCs. Similar increase in impedance was seen in untreated *Smpd1*^*−/−*^ SMCs, however, PDGF-BB treatment resulted in a marked decrease in impedance in *Smpd1*^*−/−*^ SMCs (dotted line a and b). The relative changes in the impedance during the first 16 h were summarized in Fig. 1e. The impedances of untreated *Smpd1*^+/+^ and *Smpd1*^*−/−*^ SMCs increased by 48.20 ± 18.57% and 27.45 ± 9.29%, respectively. In the presence of PDGF-BB, the impedance increased by 18.31 ± 7.03% in *Smpd1*^+/+^ SMCs, whereas the impedance decreased by 26.22 ± 7.76% in *Smpd1*^*−/−*^ SMCs. Because *Smpd1*^+/+^ and *Smpd1*^*−/−*^ SMCs exhibit similar proliferation behavior in response to PDGF-BB (Fig. 1a–c), the negative change of the impedance by PDGF-BB suggests that PDGF-BB promotes a morphological change in *Smpd1*^*−/−*^ SMCs leading to decreased cell size. The morphology was further examined by immunofluorescent staining with cytoskeleton protein α-SMA and F-actin. Indeed, PDGF-BB-treated *Smpd1*^*−/−*^ SMCs displayed obviously morphological changes with extended pseudopodia elongation and cell shrinkage (Fig. 1f). SMCs undergo apoptosis show signs of cell shrinkage, round-shaped, and detached. PDGF-BB-treated *Smpd1*^*−/−*^ SMCs exhibited extended pseudopodia and remained healthy and proliferative with no obvious increase in detached cells. Thus, the cell shrinkage is unlikely due to apoptosis. In summary, these data suggest that ASM plays a unique role in PDGF-BB-induced morphological changes but not proliferation in SMCs.

### Effects of *Smpd1* gene ablation on the vascular wall thickness and the formation of aortic sprouts

**Fig. 2:**
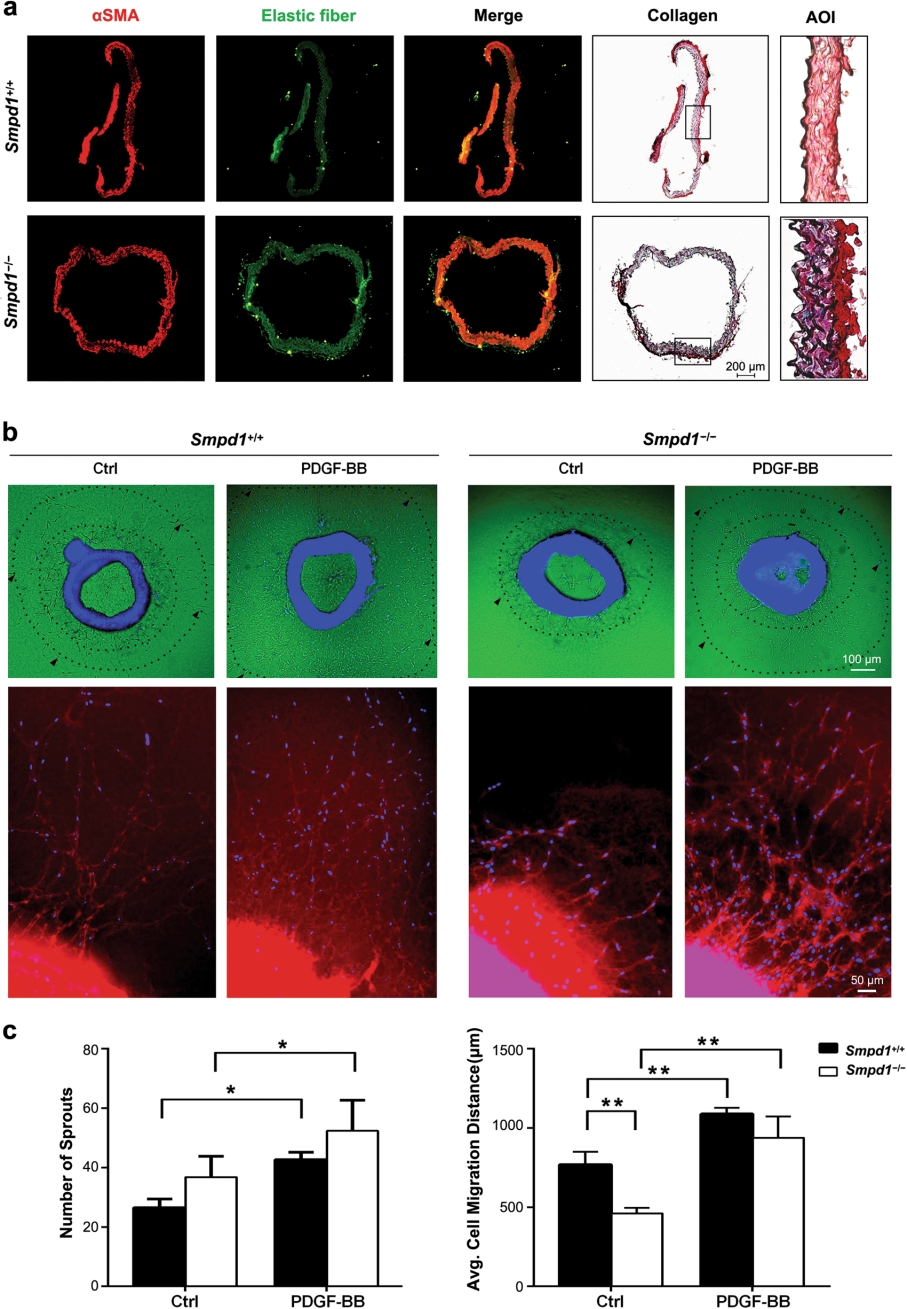
Effect of *Smpd1* gene ablation on vascular wall thickness and SMC migratory potential. **a** Immunofluorescence staining for α-SMA (red) and elastin fiber (green) of aortae from *Smpd1*^*+/+*^ and *Smpd1*^*−/−*^ mice. Collagen expression in aortae was also assessed by Sirius red staining. AOI: area of interest for enlarged region of Sirius red-collagen staining. **b**, **c** Ex vivo aortic sprout analysis of SMC migratory potential. Aortic rings from *Smpd1*^*+/+*^ and *Smpd1*^*−/−*^ mice were treated with PDGF-BB (30 ng/ml) or vehicle control for 5 days. **b** Shows the representative immunofluorescent images of aortic ring stained with α-SMA (red) and DAPI (blue). Lower panel **b** shows the enlarged images of aortic rings with migrated sprouts. Quantification data in **c** show the number of SMC sprouts emerging from the aortic ring and the total length of all branches for each outgrowth to reflect migration distance. ^*^P < 0.05 (*n* = 6)

The association of the morphological changes in *Smpd1*^*−/−*^ SMCs with vascular wall remodeling was examined in aortic explants from mice (Fig. 2a). The aortic rings were stained with α-SMA or Sirus red for collagen. *Smpd1*^+/+^ and *Smpd1*^*−/−*^ aortic rings appear to be similar in the expression of elastic fiber and vascular wall thickness. In contrast, lower α-SMA expression and higher collagen level (deep red or marron) were observed in *Smpd1*^*−/−*^ rings compared to *Smpd1*^+/+^ ring. These data suggest ASM deficiency leads to enhanced extracellular matrix production.

To determine the role of ASM in migration, the endothelium-denuded aortic rings were cultured ex vivo in Matrigel and treated with PDGF-BB. The number of sprouts that outgrown from aortic rings was quantified by analyzing cells stained with α-SMA and DAPI. The cell migration distance was measured for α-SMA^+^/DAPI^+^ cells that emigrate out of aortic explants. As shown in Fig. 2b, untreated *Smpd1*^+/+^ aortic rings showed a basal level of sprout outgrowth from aortic explants. Compared to *Smpd1*^+/+^ rings, untreated *Smpd1*^*−/−*^ rings showed significantly decreased cell migration distance but a tendency for an increase in the number of sprouts (Fig. 2c). These data suggest that, at the basal condition, similar number of SMCs can outgrow from *Smpd1*^*−/−*^ rings but these *Smpd1*^*−/−*^ SMCs migrate at much reduced speed in Matrigel. However, under PDGF-BB treatment, no significant difference was found in either the number of sprouts or cell migration in *Smpd1*^+/+^ and *Smpd1*^*−/−*^ rings (Figs. 2b, c), suggesting a maximal activation of migration potential by PDGF-BB in SMCs outgrown from *Smpd1*^+/+^ and *Smpd1*^*−/−*^ rings. Thus, ASM is involved in SMC migration at the basal condition, but it is dispensable for PDGF-BB-induced migration.

### PDGF-BB promotes myofibroblast-like phenotype transition of SMCs lacking *Smpd1* gene

**Fig. 3:**
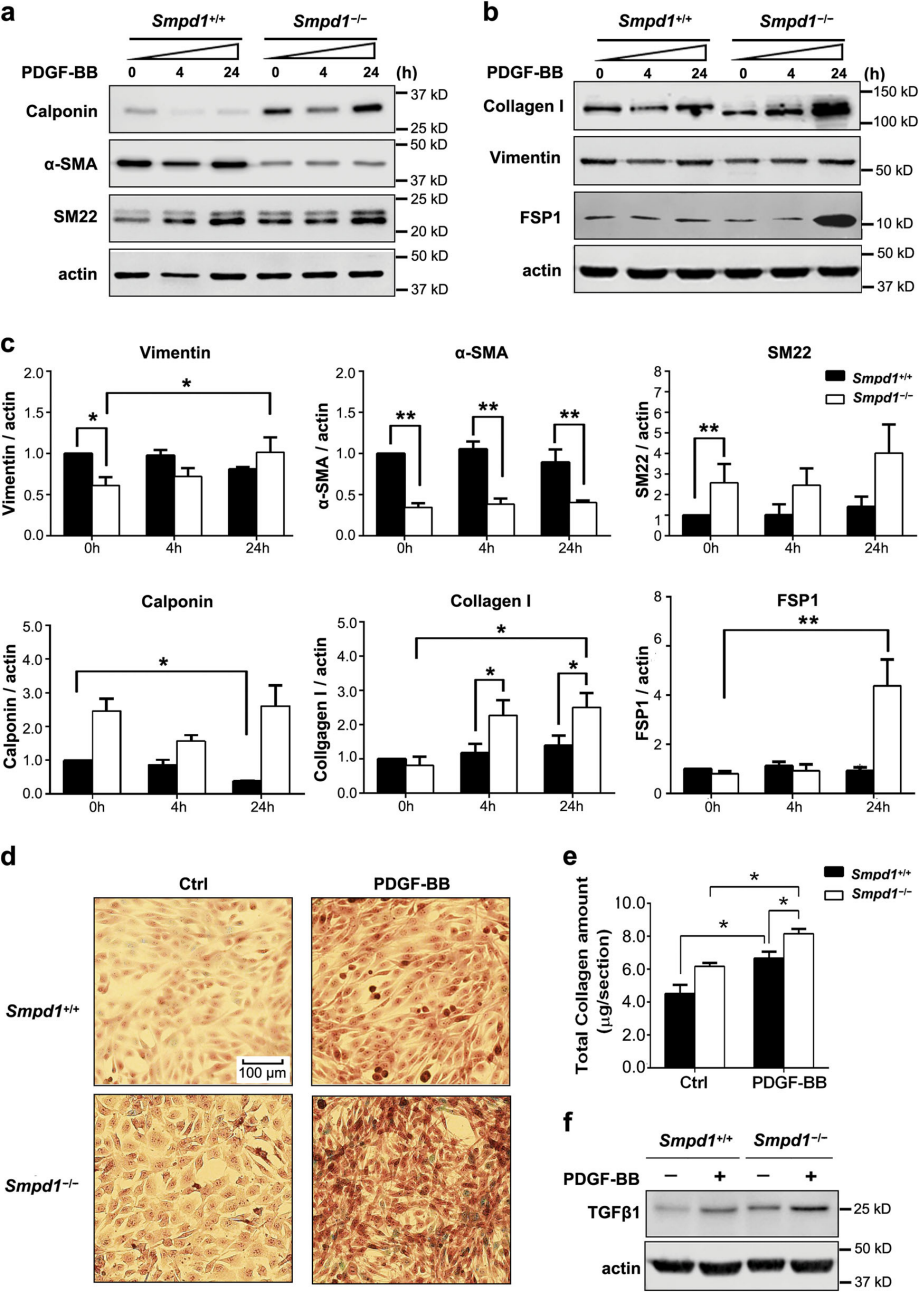
PDGF-BB promotes myofibroblast-like differentiation of *Smpd1*^*−/−*^ SMCs. SMCs were under control condition or stimulated with PDGF-BB (30 ng/ml) for the indicated time. **a** Representative western blot showing the protein expression of SM22, α-SMA, and Calponin. **b** Representative immunoblotting analysis of Vimentin, Collagen I, and FSP-1. **c** Summarized data of immunoblotting mentioned above. ^*^P < 0.05, ^**^P < 0.01 (*n* = 4). **d** Sirius Red staining of SMCs under control condition or stimulated with PDGF-BB (30 ng/ml) for 24 h. **e** Total collagen amount was examined according to Sirius red staining results. ^*^P < 0.05, ^**^P < 0.01 (*n* = 4). Two-way ANOVA with genotype and treatment as category factors. **f** Representative western blot showing the protein expression of TGF-β1 in *Smpd1*^*+/+*^ and *Smpd1*^*−/−*^ SMCs before and after PDGF-BB stimulation. The experiment was repeated at least three times with similar results

The expression of various SMC and fibroblast markers was measured and compared in between *Smpd1*^*−/−*^ and *Smpd1*^+/+^ SMCs. As shown in Fig. 3a–c, under basal condition, *Smpd1*^*−/−*^ and *Smpd1*^+/+^ SMCs had similar expression level of collagen I and fibroblast-specific protein 1 (FSP1). However, PDGF-BB treatment selectively induced upregulation of collagen I and FSP1 in *Smpd1*^*−/−*^ SMCs. The collagen I in *Smpd1*^*−/−*^ cells showed a reduced molecular weight, which may be due to deregulated post-translational modification such as glycosylation^21,22^. In addition, *Smpd1*^*−/−*^ SMCs with PDGF-BB also showed different expression pattern of SMC phenotype markers with lower α-SMA, higher calponin and SM22, but similar vimentin expression levels. The extracellular collagen deposition was confirmed by staining SMCs with Sirius red. PDGF-BB induced collagen deposition in *Smpd1*^+/+^ SMCs, which is more pronounced in *Smpd1*^*−/−*^ SMCs (Fig. 3d,e). TGF-β1 plays a key role in regulating collagen I accumulation in SMCs^23^. *Smpd1*^*−/−*^ cells showed much higher TGF-β1 expression under either basal condition or with PDGF-BB stimulation (Fig. 3f). Altogether, these results demonstrate that PDGF-BB potentially promotes the transition to myofibroblast-like phenotype characterized by elevated expression of FSP-1 and massive deposition of collagen type I.

### *Smpd1* gene ablation exacerbates PDGF-induced inflammation in SMCs

**Fig. 4:**
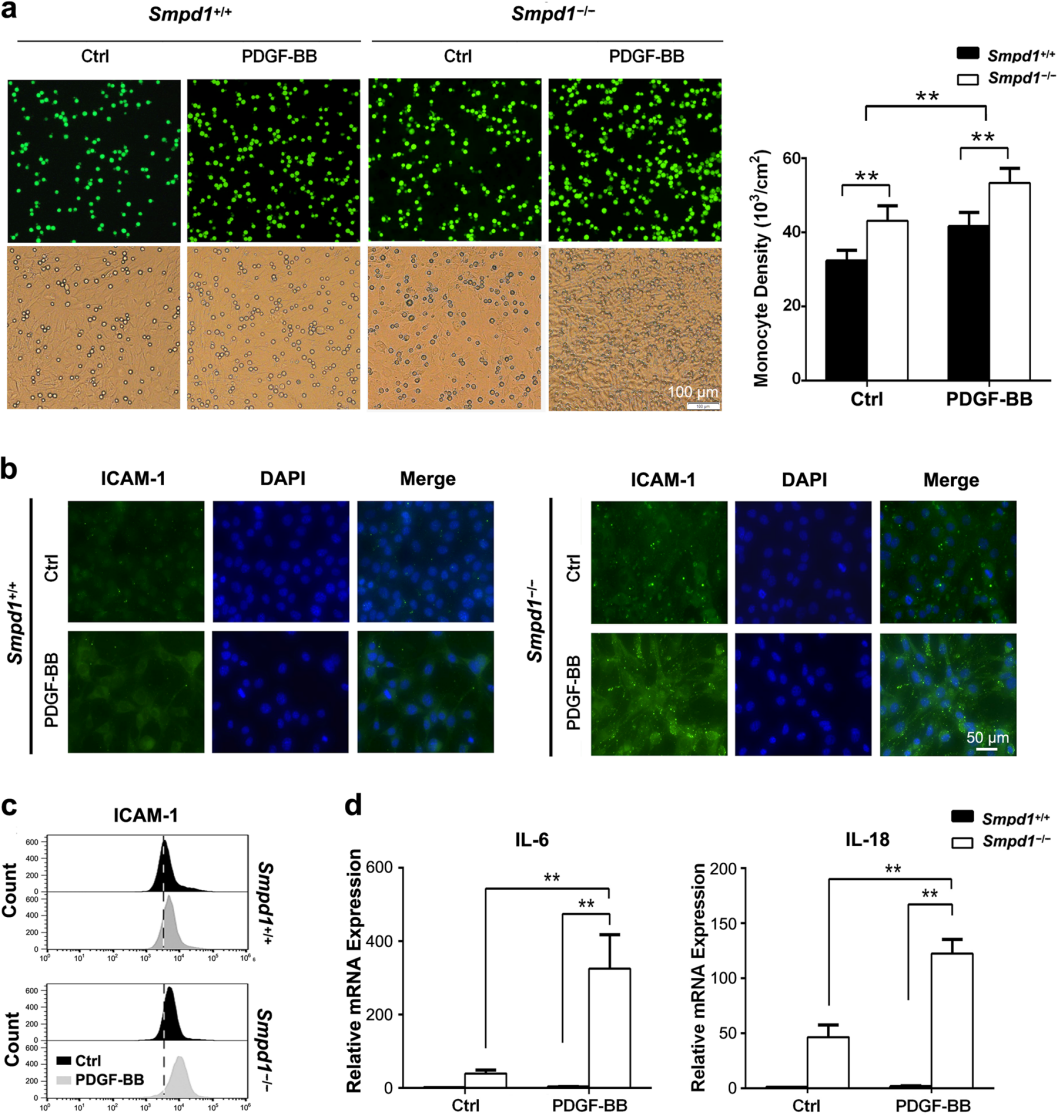
*Smpd1* gene ablation exacerbates PDGF-induced inflammation in SMCs. **a** Representative images (left) and cell quantification (right) showing the effects of ASM deficiency and PDGF-BB stimulation on monocyte (green) recruitment. SMCs were pretreated with 30 ng/ml PDGF-BB or equal amount of vehicle for 24 h and then co-cultured with calcium-AM-labeled monocytes for 30 min. Microscopic observation was conducted with a fluorescence microscope and cell counting was performed by flow cytometry by gating calcium positive cell. Magnification, ×200. *P < 0.05, **P < 0.01 (*n* = 4). Immunofluorescence (**b**) and flow cytometry (**c**) analysis of intercellular adhesion molecule-1 (ICAM-1) expression in *Smpd1*^*+/+*^ and *Smpd1*^*−/−*^ SMCs before and after PDGF-BB stimulation. **d** Real-time RT-PCR analyses of IL-6 and IL-18 mRNA expression in *Smpd1*^*+/+*^ and *Smpd1*^*−/−*^ SMCs before and after PDGF-BB stimulation. ^*^P < 0.05, ^**^P < 0.01 (*n* = 6)

Myofibroblasts are a major source of inflammation in the diseased organ by recruiting inflammatory cells and secreting inflammatory cytokines. As shown in Fig. 4a, more monocytes were found adhering to *Smpd1*^*−/−*^ SMCs than *Smpd1*^+/+^ SMCs at either the basal level or with PDGF-BB stimulation. Such enhanced recruitment of monocytes to *Smpd1*^*−/−*^ SMCs was accompanied by increased expression of intercellular adhesion molecule-1 (ICAM-1) (Fig. 4b, c). As shown in Fig. 4d, *Smpd1*^*−/−*^ SMCs had higher IL-6 and IL-18 mRNA compared to *Smpd1*^+/+^ SMCs at the basal condition, which was massively enhanced by PDGF-BB. Together, these results provide novel evidence that PDGF-BB-treated *Smpd1*^*−/−*^ SMCs gain myofibroblast-like phenotype with elevated inflammatory status.

### *Smpd1* gene ablation increases autophagosome accumulation and p62/SQSTM1 in SMCs

**Fig. 5:**
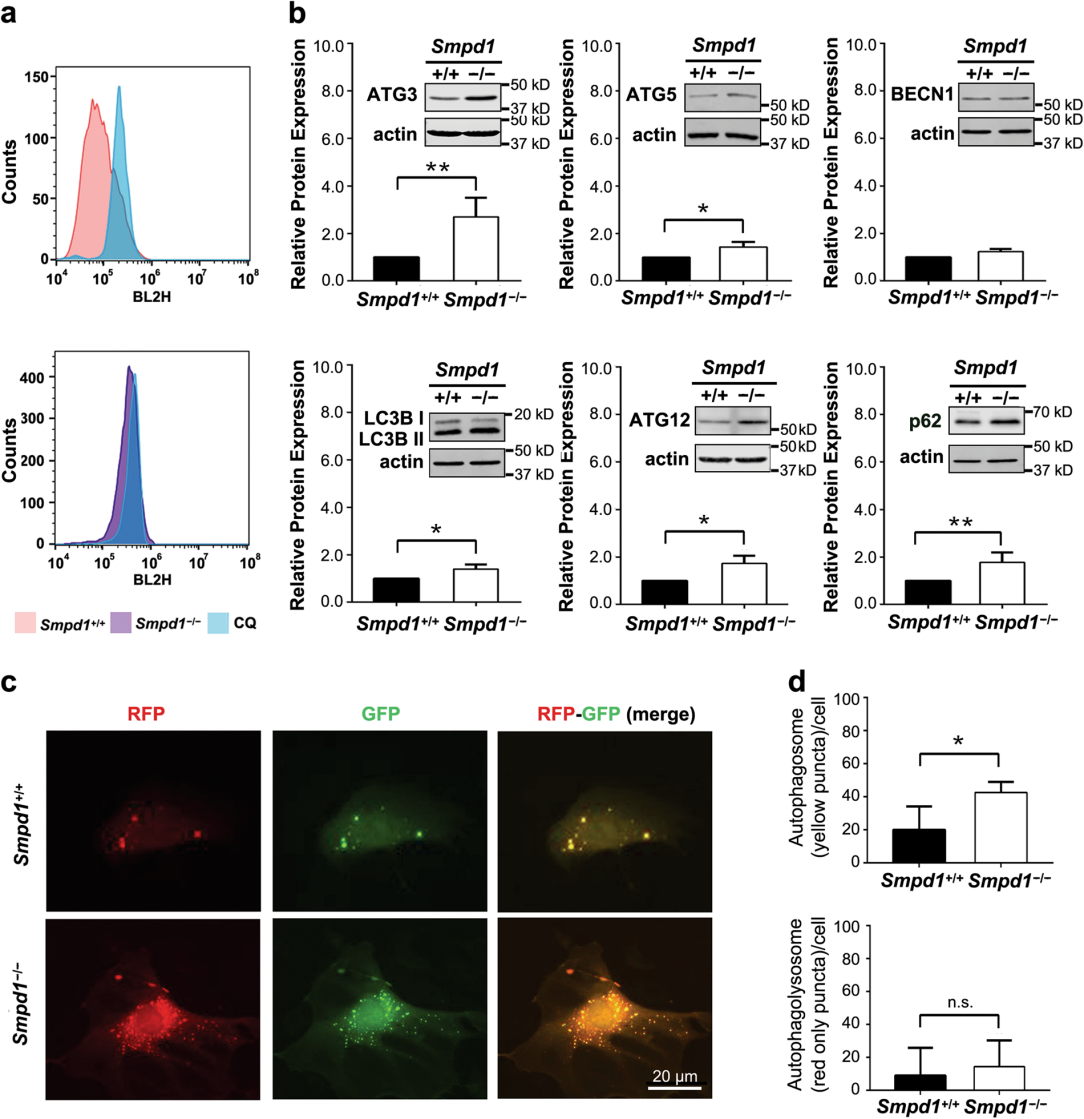
*Smpd1* gene ablation impairs autophagy flux in SMCs. **a** Shown are the representative histograms for flow cytometric analysis of CytoID Green fluorescence in *Smpd1*^*+/+*^ or *Smpd1*^*−/−*^ SMCs treated with control or CQ (chloroquine). **b** Immunoblotting and summarized data of autophagy markers in *Smpd1*^*+/+*^ and *Smpd1*^*−/−*^ SMCs under the control condition. **c** Representative fluorescent images of the tandem RFP-GFP-LC3B transfected SMCs show the formation of autophagosomes and autophagolysosomes. Autophagosomes were visualized as yellow or orange puncta (RFP-GFP-LC3B) in merged images, whereas red puncta (RFP-LC3B) in merged images represent autophagolysosomes since acidification abolishes green fluorescence. **d** Summarized data showing the number of autophagosomes (yellow/orange puncta) and autophagolysosomes (red puncta) in the merged images. ^*^P < 0.05 (*n* = 4)

Accumulating evidence has demonstrated that autophagy is critically involved in SMC phenotypic plasticity^18^. We sought to investigate the role of autophagy in myofibroblast-like transition. First, the dynamic balance of autophagosome formation and its degradation was measured by staining SMCs with CytoID. As shown in Fig. 5a, *Smpd1*^*−/−*^ SMCs had more autophagic vacuoles (as shown by increased CytoID-positive cells) than *Smpd1*^+/+^ SMCs. This increase was not abolished by chloroquine (CQ), an autophagic flux inhibitor. *Smpd1*^*−/−*^ SMCs also had higher expression of autophagosome makers including ATG 3, ATG 5, LC3B-II, ATG12 and a selective autophagy substrate p62/SQSTM1 (Fig. 5b). Thus, these results suggest that accumulation of autophagic vacuoles in *Smpd1*^*−/−*^ SMCs is related with insufficient autophagic flux but not autophagy induction. Additionally, the autophagosomes and autophagolysosomes were visualized by fluorescence microscopy in SMCs transfected with tandem RFP-GFP-LC3B plasmids. Accumulation of autophagosomes (yellow GFP^+^RPF^+^ puncta in merged image) was found in *Smpd1*^*−/−*^ SMCs, whereas no significant difference in the number of autophagolysosomes (red GFP^-^RFP^+^ puncta in merged images) was found (Fig. 5c, d). The increased ratio of autophagosomes over autophagolysosomes indicates a reduced autophagic flux in *Smpd1*^*−/−*^ SMCs. Collectively, these results suggest that SMCs lacking *Smpd1* gene exhibit reduced autophagic flux leading to the accumulation of autophagosomes and its substrate p62/SQSTM1.

### Inhibition of autophagosome formation by PDGF-BB is essential for myofibroblast-like transition in *Smpd1*^*−/−*^ SMCs

**Fig. 6:**
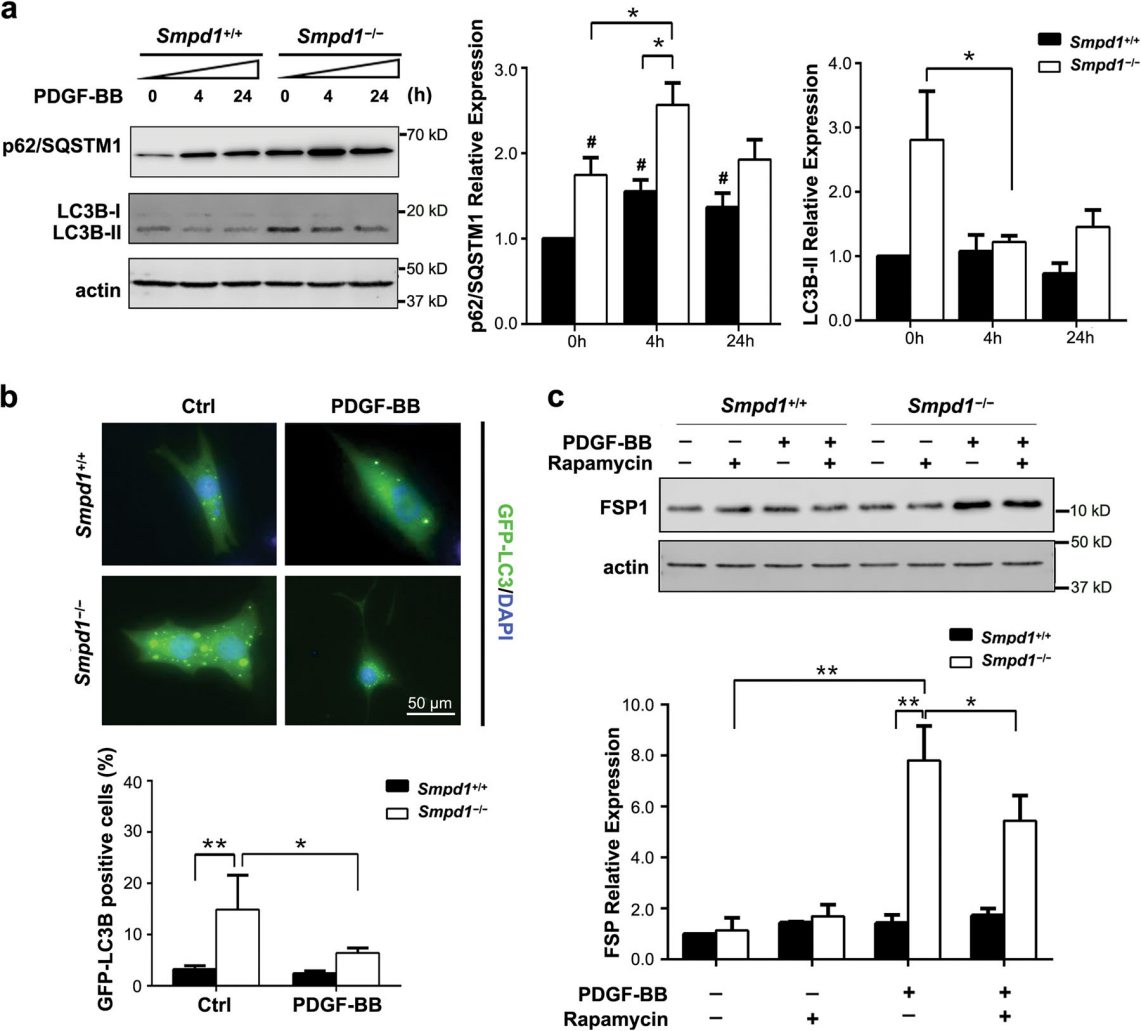
Autophagy inhibition is required for PDGF-BB-induced myofibroblast-like transition of *Smpd1*^*−/−*^ SMCs. **a** Immunoblotting and summarized data of p62/SQSTM1 and LC3B in *Smpd1*^*+/+*^ or *Smpd1*^*−/−*^ SMCs treated with control or PDGF-BB (30 ng/ml) for indicated time. *P < 0.05, **P < 0.01 (*n* = 4). **b** GFP-LC3B-expressing SMCs were seeded in 8-well Lab-Tek® Chamber Slide, allowed to adapt for 12 h, and maintained in control conditions or exposed to PDGF-BB (30 ng/ml) for 4 h. Representative images and quantitative data are reported. *P < 0.05, **P < 0.01 (*n* = 6). **c** Immunoblotting and summarized data of FSP-1 in *Smpd1*^*+/+*^ or *Smpd1*^*−/−*^ SMCs treated with control or PDGF-BB (30 ng/ml) in the presence or absence of rapamycin (50nmol/l) for indicated time. *P < 0.05, **P < 0.01 (*n* = 6).

As shown in Fig. 6a, PDGF-BB treatment significantly enhanced p62/SQSTM1 expression in *Smpd1*^*−/−*^ SMCs compared to *Smpd1*^+/+^ cells. Moreover, PDGF-BB reduced LC3B-II in *Smpd1*^*−/−*^ SMCs but not in *Smpd1*^+/+^ SMCs (Fig. 6a). Consistently, PDGF-BB also reduced LC3B in *Smpd1*^*−/−*^ SMCs transfected with GFP-LC3 plasmid (Fig. 6b). LC3B-II expression reflects the dynamic balance of autophagosome biogenesis and its turnover via autophagic flux. Thus, these results suggest that in *Smpd1*^*−/−*^ SMCs, impaired autophagic flux leads to p62/SQSTM1 accumulation, which was further exacerbated by PDGF-BB as it reduces the autophagosome biogenesis. Inhibition of mTOR by rapamycin is commonly used to stimulate autophagy by inducing autophagosome formation in mammalian cells. Interestingly, rapamycin partially attenuated PDGF-BB-induced FSP-1 expression in *Smpd1*^*−/−*^ SMCs (Fig. 6c). As a comparison, neither rapamycin nor its combination with PDGF-BB had any effect on FSP-1 in *Smpd1*^+/+^ SMCs. These results suggested that restoration of autophagy may at least partially prevent PDGF-BB-induced myofibroblast-like transition in *Smpd1*^*−/−*^ SMCs.

### Sustained Akt activation is essential for PDGF-BB-induced myofibroblast-like transition of *Smpd1*^*−/−*^ SMCs

**Fig. 7:**
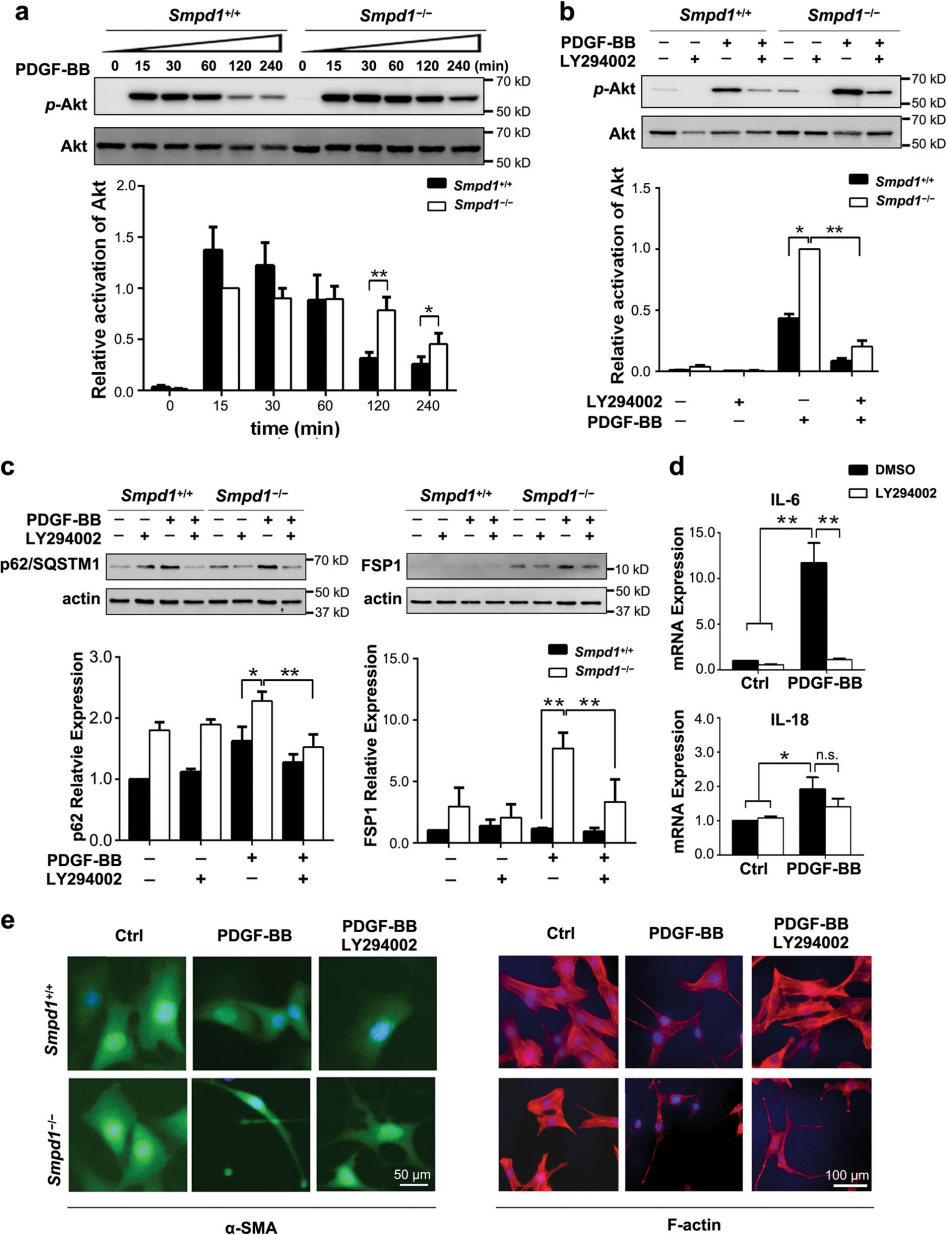
Sustained Akt activation is required for in PDGF-BB induced myofibroblast transition of *Smpd1*^*−/−*^ SMCs. **a** Representative images and quantitative data show the effects of PDGF-BB (30 ng/ml) on the phosphorylation of Akt and Erk-1/2 in in *Smpd1*^*+/+*^ or *Smpd1*^*−/−*^ SMCs. *P < 0.05, **P < 0.01 (*n* = 4). **b**, **c** Representative images and quantitative data show the effect of Akt inhibitor LY294002 (50μmol/l) on PDGF-BB-induced changes in Akt phosphorylation, p62/SQSTM1, and FSP-1 in *Smpd1*^+/+^ or *Smpd1*^−/−^ SMCs. SMCs were treated with or without PDGF-BB (30 ng/ml) for 2 h for Akt phosphorylation or for 24 h for analysis of p62/SQSTM1 and FSP-1. *P < 0.05, **P < 0.01 (*n* = 4). **d** Real-time RT-PCR analyses of IL-6 and IL-18 mRNA expression in *Smpd1*^−/−^ SMCs treated with or without PDGF-BB (30 ng/ml) for 24 h in the presence or absence of LY294002 (50μmol/l). ^*^P < 0.05, ^**^P < 0.01 (*n* = 6). **e** Representative immunofluorescence images of *Smpd1*^+/+^ or *Smpd1*^−/−^ SMCs treated with or without PDGF-BB (30 ng/ml) in the presence or absence of LY294002 (50μmol/l) for 24 h. The experiment was repeated at least three times

Multiple survival signaling pathways such as Akt and Erk1/2 are activated by PDGF in SMCs, both of which can activate mTOR signaling that inhibits autophagy induction^24^. As shown in Fig. 7a, a more sustained activation of Akt was induced by PDGF-BB in *Smpd1*^*−/−*^ SMCs compared to that in *Smpd1*^+/+^ SMCs. In contrast, there was no significant difference in Erk1/2 activation between *Smpd1*^+/+^ and *Smpd1*^*−/−*^ cells (data not shown). LY294002, an Akt inhibitor, efficiently suppressed PDGF-BB-induced Akt activation in both *Smpd1*^+/+^ and *Smpd1*^*−/−*^ cells (Fig. 7b). More importantly, in *Smpd1*^*−/−*^ SMCs, the Akt suppression by LY294002 was accompanied by significant reduction of PDGF-BB-induced p62/SQSTM1 accumulation and FSP-1 (Fig. 7c). Interestingly, LY294002 markedly attenuated PDGF-BB-induced mRNA level of IL-6 but not IL-18 in *Smpd1*^*−/−*^ SMCs (Fig. 7d). Further, LY294002 markedly reversed morphological changes by PDGF-BB in *Smpd1*^*−/−*^ SMCs as evidenced by the reduction of prolonged cytoplasmic projections and restoration of cell size (Fig. 7e). Altogether, these results raise a possibility that sustained Akt activation inhibits autophagosome formation and thereby contributes to myofibroblast transition.

### Akt-p62/SQSTM1 axis contributes to PDGF-induced myofibroblast transition in *Smpd1*^*−/−*^ SMCs

**Fig. 8:**
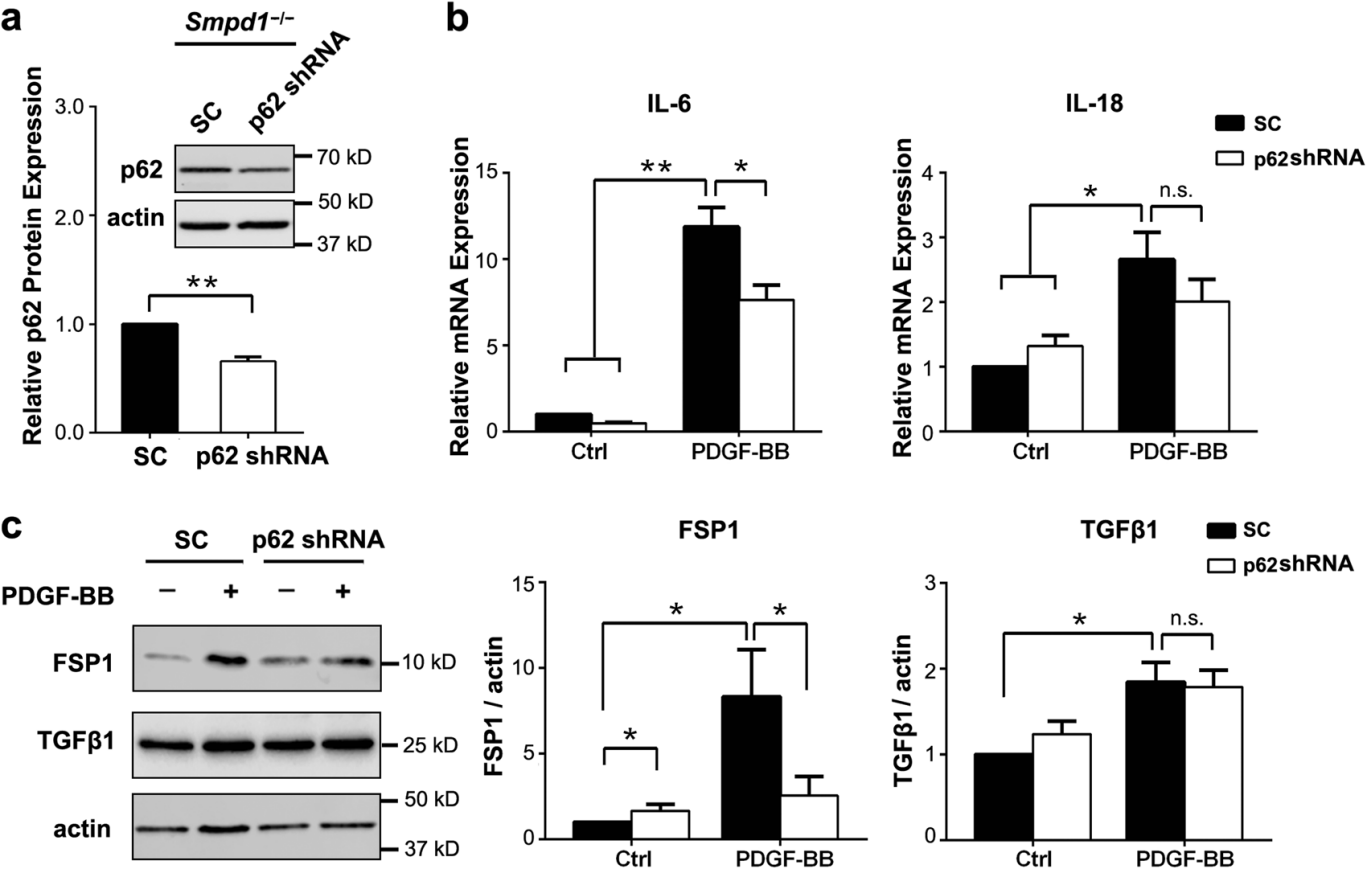
Akt-p62/SQSTM1 axis contributes to PDGF-BB induced myofibroblast-like phenotype transition of *Smpd1*^*−/−*^ SMCs. Smpd1^*−/−*^ SMCs were infected with p62/SQSTM1 shRNA or scramble shRNA lentiviral particles and then treated with PDGF-BB (30  ng/ml) for 24  h. **a** Representative immunoblotting and summarized data show the protein expression of p62/SQSTM1 in *Smpd1*^*−/−*^ SMCs infected with p62/SQSTM1 shRNA or scramble shRNA (SC) lentiviral particles. *P < 0.05, **P < 0.01 (*n* = 3). **b** Real-time RT-PCR analyses of IL-6 and IL-18 mRNA expression in PDGF-BB or vehicle treated *Smpd1*^−/−^ SMCs with p62/SQSTM1 silencing or not. ^*^P < 0.05, ^**^P < 0.01 (*n* = 6). **c** Representative immunoblotting and summarized data show the effects of p62/SQSTM1 shRNA on PDGF-BB-induced protein expression of FSP-1 and TGF-β1 in *Smpd1*^−/−^ SMCs

As shown in Fig. 8a, b, p62/SQSTM1 gene silencing markedly inhibited PDGF-BB-induced expression of IL-6 but not IL-18 in *Smpd1*^*−/−*^ SMCs. p62/SQSTM1 gene silencing almost diminished FSP-1 expression induced by PDGF-BB but had no effect on TGF-β1 (Fig. 8c). FSP-1 gene expression was more drastically reduced by p62/SQSTM1 silencing compared to IL-6, which might indicate that FSP-1 gene requires a higher threshold of p62/SQSTM1 level for its gene induction. These findings suggest that Akt-p62/SQSTM1 pathway contributes to the myofibroblast transition of *Smpd1*^*−/−*^ SMCs upon PDGF-BB stimulation.

### Involvement of Nrf2 in PDGF-BB-induced IL-6 production in *Smpd1*^*−/−*^ SMCs

**Fig. 9:**
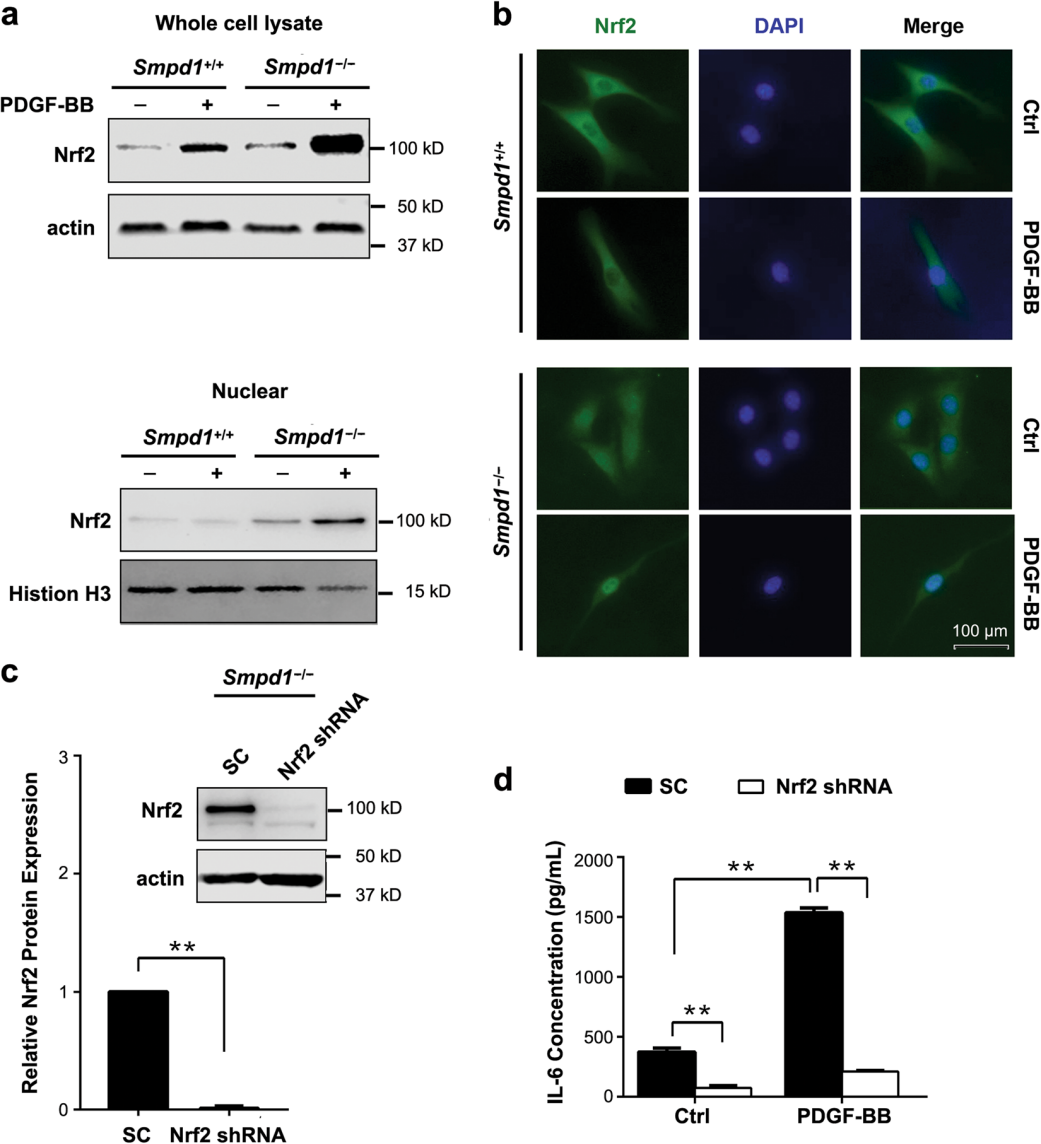
Inhibition of Nrf2 activation attenuates PDGF-BB induced IL-6 production in *Smpd1*^*−/−*^ SMCs. **a** Immunoblots show the expression of Nrf2 in whole-cell lysate or nuclear extract of *Smpd1*^*+/+*^ and *Smpd1*^*−/−*^ SMCs treated with or without PDGF-BB (30 ng/ml) for 24 h. The experiment was repeated three times. **b** Immunofluorescence images show the nuclear translocation of Nrf2 (green) in *Smpd1*^*+/+*^ and *Smpd1*^*−/−*^ SMCs treated with or without PDGF-BB (30 ng/ml) for 24 h. Nuclei were stained with DAPI (blue). The experiment was repeated three times. **c** Immunoblots show the efficiency of Nrf2 shRNA or scramble shRNA (SC) on Nrf2 expression in *Smpd1*^*−/−*^ SMCs. **P < 0.01 (*n* = 3). **d** IL-6 release in *Smpd1*^−/−^ SMCs. *Smpd1*^−/−^ SMCs were transduced with Nrf2 shRNA or scramble shRNA and then treated with or without PDGF-BB (30 ng/ml) for 24 h. IL-6 was measured by ELISA. ^*^P < 0.05, ^**^P < 0.01 (*n* = 6)

Recently studies demonstrated a role of p62/SQSTM1 in linking autophagy and Nrf2 signaling. Accumulated p62/SQSTM1 causes a non-canonical activation of Nrf2 leading to cell proliferation and differentiation^25^. As shown in Fig. 9a, more significant Nrf2 upregulation was observed in *Smpd1*^*−/−*^ SMCs treated with PDGF-BB compared to *Smpd1*^*+/+*^ SMCs, especially in the nuclear fraction. Consistently, PDGF-BB induces a more pronounced nuclear translocation of Nrf2 in *Smpd1*^*−/−*^ SMCs than *Smpd1*^*+/+*^ SMCs (Fig. 9b). Further, Nrf2 gene silencing significantly decreased Nrf2 expression and abolished PDGF-BB induced IL-6 production in *Smpd1*^*−/−*^SMCs (Fig. 9c, d). Collectively, these results demonstrated that Nrf2 plays a role in *Smpd1*^*−/−*^ SMCs differentiation.

## Discussion

In the present study, we demonstrated that PDGF-BB induced a myofibroblast-like phenotype in *Smpd1*^*−/−*^ SMCs, which is distinct from PDGF-BB-induced “canonical” synthetic phenotype in *Smpd1*^+/+^ SMCs. This unique myofibroblast-like “non-canonical” synthetic phenotype in *Smpd1*^*−/−*^ SMCs is characterized by: (1) morphological changes with extended pseudopodia elongation and cell shrinkage; (2) elevated expression of fibroblast marker FSP-1; (3) enhanced extracellular deposition of collagens; (4) massive production of inflammatory cytokines. Our findings further demonstrated that ASM participates in PDGF-BB-induced SMC myofibrogenic switching by modulating Akt-autophagy-p62/SQSTM1 axis.

PDGF-induced SMC proliferation and migration are essential processes in vascular disorders such as atherosclerosis^1,26^. Our data showed that PDGF-BB induced similar changes in cell cycle, cyclin D1 expression, or cell numbers in between *Smpd1*^*−/−*^ and *Smpd1*^+/+^ cells. Further, ASM deficiency had no effect on PDGF-BB-induced outgrowth of sprouts from aortic explants. Our data, thus, reveal that PDGF-BB-treated *Smpd1*^*−/−*^ SMCs remain proliferative and migratory, which resembles “canonical” synthetic phenotype. PDGF-BB induced obvious morphological changes in *Smpd1*^*−/−*^ SMCs with extended pseudopodia elongation and cell shrinkage, whereas *Smpd1*^+/+^ SMCs have a spindle-shaped appearance. We also found that PDGF-BB-treated *Smpd1*^*−/−*^ SMCs rapidly reduced their cell size as reflected by decreased cell impedance, particularly in the early stage of PDGF-BB stimulation. Such decrease in cell size raises a possibility that dedifferentiated *Smpd1*^*−/−*^ SMCs convert readily to a myofibroblastic-like phenotype that is different from “canonical” synthetic phenotype observed in *Smpd1*^+/+^ SMCs.

So far, there is no specific marker to discriminate myofibroblasts from fibroblasts or SMCs. Myofibroblasts have a phenotype intermediate between fibroblasts and SMCs. The myofibroblast-like phenotype may possess the differentiation markers of fibroblasts and SMCs. It is necessary to compare multiple SMC differentiation marker and fibroblast marker between the morphologically different phenotypes. Several SMC markers (α-SMA, calponin, SM22) are differently expressed in myofibroblast-like *Smpd1*^*−/−*^ SMCs compared to “canonical” synthetic *Smpd1*^+/+^ SMCs. Among them, the expression level of α-SMA is lower in *Smpd1*^*−/−*^ SMCs than *Smpd1*^+/+^ SMCs. Although α-SMA is a classic SMC marker and not expressed in endothelial cells or fibroblasts, it is also a commonly used marker to identify myofibroblast from fibroblast in fibrotic diseases^27^. The SMC dedifferentiation is associated with decreased expression of α-SMA^28^. Therefore, the lower expression of α-SMA in myofibroblast-like *Smpd1*^*−/−*^ SMCs suggest a more dedifferentiated state in these cells than “canonical” synthetic *Smpd1*^+/+^ SMCs. Interestingly, myofibroblastic-like *Smpd1*^*−/−*^ SMCs have increased expression of contractile markers calponin and SM22. Calponin is a calmodulin, F-actin and tropomyosin binding protein that is involved in smooth muscle contraction^29^. SM22 is a transformation and shape-change sensitive actin cross-linking/gelling protein^30^. As calponin and SM22 are contraction and shape-related proteins, the increases in calponin and SM22 may be associated with the alteration in contractility and/or morphological changes during myofibroblast-like transition. Further, myofibroblast-like *Smpd1*^*−/−*^ SMCs induced by PDGF-BB are highly expressed in FSP-1, have enhanced collagen deposition and TGF-β1 secretion, and demonstrate increased adhesion to monocytes and massive production inflammatory cytokines (IL-6 and IL-18), all of which are commonly found in activated myofibroblasts^31^. To our knowledge, our findings provide the first evidence that PDGF-BB promoted a myofibroblastic transition in *Smpd1*^*−/−*^ SMCs.

Recent studies demonstrated that moderately enhanced autophagy in the vasculature protects against progressive atherosclerosis or restenosis^32^. Autophagy blunts proliferation in SMCs under various atherogenic stimuli such as thrombin and advanced glycation end products^33,34^. Enhanced autophagy by either statins or rapamycin helps maintain vascular smooth muscle in contractile phenotype and inhibits their proliferation^18,32^. ASM-deficient SMCs exhibits reduced autophagic flux that contributes to enhanced proliferation by 7-ketocholesterol^17^. These previous studies suggest that ASM-mediated autophagy plays a protective role in SMCs by maintaining contractile phenotype. PDGF-BB increases autophagosome induction and inhibition of autophagy prevents PDGF-BB-induced synthetic phenotype transition in rat aortic SMCs, which suggest that autophagy is critical for attaining the synthetic phenotype^35,36^. However, ASM deficiency had no effect on PDGF-BB-induced proliferation in SMCs despite these cells had reduced autophagic flux. Moreover, PDGF-BB caused an inhibition of autophagosome biogenesis in *Smpd1*^*−/−*^ SMCs, which was concomitant with augmented p62/SQSTM1 expression. Interestingly, reactivation of autophagy by rapamycin, an inhibitor of mTOR, markedly attenuated PDGF-BB-induced FSP-1 expression in *Smpd1*^*−/−*^ SMCs. Therefore, our findings support the view that PDGF-BB-induced autophagosome inhibition and defective autophagic flux act synergistically to induce or promote myofibroblastic transition in *Smpd1*^*−/−*^ SMCs.

Moreover, this study reveals that sustained activation of Akt signaling by PDGF-BB leads to the inhibition of autophagosome biogenesis, which contributes to enhanced p62/SQSTM1 accumulation in *Smpd1*^*−/−*^ SMCs. PDGF-BB binds with PDGF receptors leading to activation of pathways such as Akt and mitogen-activated protein kinase Erk1/2 cascades^37^. Both Akt and Erk1/2 pathways regulate autophagy signaling as well as proliferation of mammalian cells^38,39^. In *Smpd1*^*−/−*^ SMCs, PDGF-BB induced a sustained activation of Akt but not Erk1/2 signaling. Ceramide, the hydrolysis product of sphingomyelin by ASM, activates phosphatase 2 A (PP2A), which dephosphorylates and inhibits Akt^40^. The sustained Akt activation in *Smpd1*^*−/−*^ SMCs could be due to the absence of ceramide-PP2A axis. Targeting Akt signaling by LY294002 attenuated PDGF-BB-induced expression of p62/SQSTM1 and FSP-1, reduction of cell size, and production of IL-6 in *Smpd1*^*−/−*^ SMCs. Altogether, our findings indicate a causative role of sustained Akt activation in the inhibition of autophagosome biogenesis, which importantly contributes to myofibroblastic transition in *Smpd1*^*−/−*^ SMCs.

The present study further investigated the molecular mechanisms linking diminished autophagy signaling and myofibroblastic transition. p62/SQSTM1 participates in epithelial-to-myofibroblast transition (EMT) that podocytes lose epithelial features and acquire myofibroblast features such as increased expression of FSP-1 and α-SMA^41^. Similarly, the present study for the first time demonstrated a critical role of the autophagy-p62/SQSTM1 axis in several key characteristics of myofibroblast-like phenotype including FSP-1 and IL-6 expression. However, PDGF-BB-induced TGFβ1 expression and IL-18 production are independent of p62/SQSTM1 signaling. Therefore, our data suggest that the myofibroblastic transition of *Smpd1*^*−/−*^ SMCs is through both p62SQSTM1-dependent and -independent pathways. p62/SQSTM1 may link to several signaling pathways such as cyclin-dependent kinase 1 (CDK1)-mediated p62 phosphorylation, Nrf2-mediated redox signaling, and NF-ҡB-dependent transcriptional regulation^42,43^. We demonstrated that PDGF-BB caused abnormal Nrf2 activation in *Smpd1*^*−/−*^ SMCs, which contributes to IL-6 production. Therefore, these results suggest that Nrf2 may be a downstream target of p62/SQSTM1 to induce IL-6. Future investigation is needed to decipher the precise mechanism by which autophagy-p62/SQSTM1 axis or p62/SQSTM1-independent pathway triggers the myofibroblastic transition.

**Fig. 10:**
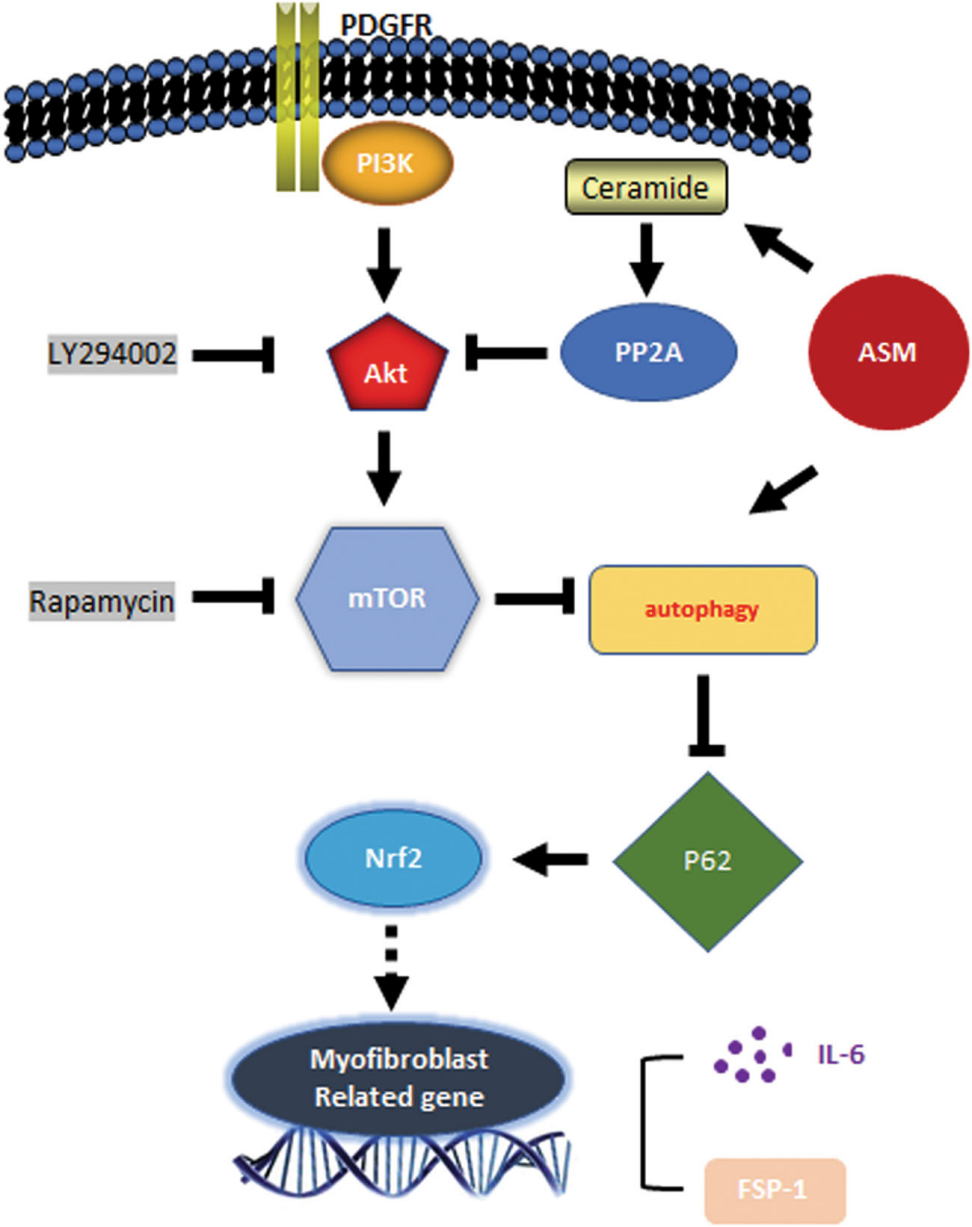
**Proposed mechanism by which ASM deficiency accelerates myofibroblast-like phenotype transition in** ***Smpd1***^*−/−*^ upon PDGF-BB stimulation (see discussion for detail description)

In summary, our work highlights the importance of ASM-autophagy cascade in myofibroblastic transition. As depicted in Fig. 10, in our system, ASM modulates both autophagosome biogenesis and its lysosome-mediated autophagic flux pathway. Ceramide produced by ASM activity activates PP2A and limits PDGF-BB-induced Akt and mTOR activation. ASM deficiency leads to reduced ceramide production, deactivation of PP2A, and consequent long-term activation of Akt-mTOR signaling. Such long-term Akt-mTOR activation suppresses autophagosome biogenesis. In another aspect, ASM activity is needed for autophagic flux. ASM deficiency reduces autophagic flux leading to delayed turnover of autophagosomes. Therefore, in situation of ASM deficiency, reduced autophagosome biogenesis and turnover synergistically cause diminished autophagy signaling leading to exacerbated accumulation of p62/SQSTM1 that may trigger its downstream activation of myofibroblastic reprogramming such as expression of FSP-1 and IL-6 genes. These results provide novel insights into a new therapeutical intervention for preventing SMC-myofibroblast transition in vascular diseases such as atherosclerosis.

## Materials and methods

### Mice

ASM-deficient (*Smpd1*^*-/-*^; *Smpd1* is the gene symbol for ASM gene *sphingomyelin phosphodiesterase 1*) and wild-type (*Smpd1*^*+/+*^) mice were used in the present study as we described previously^44,45^. All experimental protocols were reviewed and approved by the Animal Care Committee of the University of Houston. All animals were provided with standard rodent chow and water ad libitum in a temperature-controlled room.

### Antibodies and reagents

Mouse recombinant PDGF-BB (SRP3229), Direct Red 80 (365548), picric acid (197378), Fast green FCF (F7252), and anti-α-smooth muscle actin mouse mAb (C6189) were purchased from Sigma-Aldrich (St. Louis, MO, USA). Antibodies against mouse Collagen I (ab21286), Vimentin (ab92547), α-smooth muscle actin (ab5694), ICAM1(ab119871), SM22 alpha (ab14106), P62/SQSTM1 (ab109012) were from Abcam (Cambridge, UK). Anti-mouse Atg3 (#3415), Atg5 (#12994), Beclin-1 (#3495), LC3A/B (#12741), Atg12 (#4180), phospho-Akt (#4060), Akt (#9272), phospho-p44/42 MAPK (Erk1/2) (#4370), p44/42 MAPK (Erk1/2) (#4695), phospho-AMPKα (Thr172) (#2535), AMPKα (#2532), phospho-mTOR (Ser2448) (#5536), mTOR (#2983), β-actin (#8457), TGF-β, and Fibroblast-specific protein 1 (13018) were from Cell Signaling Technology (Danvers, MA, USA). Antibody specific to Cyclin D1 (554181) was obtained from BD Pharmingen. IRDye® 800CW Goat anti-Rabbit and IRDye® 680CW goat anti-mouse secondary antibodies were from LI-COR (Lincoln, NE, USA). Alexa Fluor^®^ 488 and Alexa Fluor^®^ 555 conjugated secondary antibodies, ActinRed™ 555 ReadyProbes® Reagent (R37112), Premo™ Autophagy Tandem Sensor GFP-LC3B Kit (P36239), FxCycle™ PI/RNase Staining Solution (F10797) and Fast-red were purchased from Thermo Fisher Scientific (Waltham, MA, USA). CYTO-ID® Autophagy Detection Kit 2.0 was obtained from Enzo Life Sciences (New York, NY, USA). Sqstm1-mouse shRNA lentiviral particles (TL515047V) were prepared by Origene (Rockville, MD, USA).

### Primary culture of SMCs

Mouse coronary arterial SMCs were isolated as previously described^46^. Six-week-old male C57BL/6J ASM-deficient (*Smpd1*^*−/−*^) mice and their wild-type littermates (*Smpd1*^*+/+*^) were used in the present study. In brief, mice were deeply anesthetized with intraperitoneal injection of pentobarbital sodium (25 mg/kg). The heart was excised with an intact aortic arch and immersed in a petri dish filled with ice-cold Krebs–Henseleit solution. A 25-gauge needle filled with Hanks’ buffered saline solution was inserted into the aortic lumen opening while the whole heart remained in the ice-cold buffer solution. The opening of the needle was inserted deep into the heart close to the aortic valve. The needle was tied in place with the needle tip as close to the base of the heart as possible. The infusion pump was started with a 20-ml syringe containing warm HBSS through an intravenous extension set at a rate of 0.1 ml/min for 15 min. HBSS was replaced with warm enzyme solution (1 mg/ml collagenase type I, 0.5 mg/ml soybean trypsin inhibitor, 3% bovine serum albumin, and 2% antibiotic), which was flushed through the heart at a rate of 0.1 ml/min. Perfusion fluid was collected at 30, 60, and 90-min intervals. At 90 min, the heart was cut with scissors, and the apex was opened to flush out the cells that collected inside the ventricle. The fluid was centrifuged at 1000 rpm for 10 min, the cell-rich pellets were mixed with the media described below, and the cells were plated on 2% gelatin-coated six-well plates and incubated in 5% CO_2_ at 37 °C. Advanced Dulbecco’s modified Eagle’s medium (DMEM) with 10% fetal bovine serum, 10% mouse serum, and 2% antibiotics were used for isolated SMCs. The identification of SMCs was based on positive staining by anti-α-actin antibody and the SMC morphology. The medium was replaced 3 days after cell isolation and then once or twice each week until the cells grew to confluence. All studies were performed with cells of passage of 3–6.

### Electric cell-substrate impedance sensing (ECIS) assay

ECIS can detect and quantify morphology changes in the sub-nanometer to micrometer range. The impedance is related to the number of cells covering the electrode, the morphology of the cells, and the nature of the cell attachment. The changes in cell function may alter its morphology by decreasing its size and thereby decrease the impedance. The ECIS® Zθ (theta) (Applied BioPhysics, USA) was used to monitor cell behavior according to the manufacturer’s instructions. Briefly, before starting ECIS measurements, 200 μl of complete medium was placed in each well and allowed to equilibrate in the incubator for 30 min. Then 2 × 10^5^ *Smpd1*^*+/+*^ and *Smpd1*^*−/−*^ SMCs were separately seeded in 8W10E + plates containing gold film surface electrodes in DMEM supplemented with 10% fetal bovine serum (FBS). When cells attached and spread to the electrodes of ECIS Arrays, they act as insulators increasing the impedance. As cells grow and cover the electrodes, the current is impeded in a manner related to the number of cells covering the electrode, the morphology of the cells and the nature of the cell attachment. When cells are stimulated to change their function, the accompanying changes in cell morphology alter the impedance. The cells were monitored noninvasively under current pulses 40 kHz with 600 s interval within 72 h. The data generated is impedance versus time.

### Immunoblotting

Cells were lysed in Laemmli sample buffer (Bio-Rad, 161–0737) containing β-mercaptoethanol (Sigma-Aldrich, M3148) and boiled for 10 min at 95 °C. 30 μg of total proteins were separated by 8–12% sodium dodecyl sulfate-polyacrylamide gel electrophoresis. The proteins of these samples were then electrophoretically transferred at 100 V for 1 h onto a PVDF membrane (Bio-Rad, USA). The membrane was blocked with 5% non-fat milk in Tris-buffered saline-Tween 20. After washing, the membrane was probed with primary antibody as indicated according to the manufacturer’s instructions. After washing, the membranes were then incubated with IRDye^®^ donkey anti-mouse or anti-rabbit fluorescence-conjugated secondary antibodies and bands were visualized and analyzed by LI-COR^®^ Odyssey Fc System.

### Cell cycle analysis

SMCs were plated into 6 well plates at a density of 1 × 10^6^ cells/ml and incubated for 2 h treatment with 2% FBS. Then the medium was replaced with fresh medium containing PDGF-BB (30 ng/ml) and incubated for 24 h at 37 °C. The cells were harvested washed with ice-cold PBS for three times and then fixed with 70% ethanol for 30 min at 4 °C. Subsequently, FxCycle™ PI/RNase Staining Solution (molecular probes, Life Technologies) was added to the fixed cells. In total, 1 × 10^4^ cells were analyzed by flow cytometry (Attune NxT acoustic focusing cytometer, Life Technologies, USA). All experiments were repeated three times and data analysis was recorded using AttuneR NxT software.

### Collagen staining and quantification

Total collagen amount was assayed using Sirius red staining as previously described^47^. Briefly, the Picro-Sirius red stain solution (0.1% direct red 80 plus 0.1% fast green FCF in saturated aqueous picric acid) was freshly prepared before use. For in vitro culture cell layers, 5 × 10^5^ SMCs were cultured in 24-well plate and then treated with PDGF-BB (30 ng/ml) or vehicle alone for 24 h. Then removed the culture medium and washed the cells with PBS for three times. The cell layers were completely immersed and fixed with Kahle fixative and incubated for 10 min at room temperature. After being washed with PBS for three times, cells were incubated with dye solution for 30 min at room temperature. Carefully removed the dye solution and rinsed the stained cell layers with 0.5 ml distilled water repeatedly until the fluid is colorless. For paraffin-embedded tissue sections, deparaffinized and hydrated tissue sections were covered with adequate Picro-Sirius red solution and incubated for 60 minutes at room temperature. Then rinsed slide quickly in 10 dips of distilled water and quickly dehydrated and mounted in xylene. To calculate the amount of collagen, the following formulas were used: Collagen (μg/section) = [OD_540_ value–(OD_605_ value × 0.291)]/0.0378; Non-collagenous proteins (μg/section) = OD_605_ value/0.00204.

### Monocyte retention and quantification

Mouse macrophage J774 cells were preincubated in DMEM containing 2% FBS and 5μmol/l fluorescent calcium-AM (C3100MP, Thermo Fisher Scientific, USA) at 37 °C for 30 min. Fluorescently labeled cells were washed twice to remove unincorporated dye and were then resuspended in DMEM containing 2% FBS. Loaded monocytic cells (1 × 10^5^) were added to each well of SMCs and incubated at 37 °C. After 30 min, unbound monocytes were withdrawn and SMC layers with attached monocytes were gently washed twice with DMEM. Fluorescence imaging (excitation 485 nm, emission 535 nm) was performed using Olympus IX73 imaging system. For cell quantification, the number of recruited J774.2 cells were assessed by flow cytometry to quantify green calcium-AM positive cells.

### Quantitative real-time PCR

Total RNA was isolated using the Aurum Total RNA Mini Kits (Bio-Rad, Cat. #732-6820, USA) according to the manufacturer’s instructions. cDNA was generated from the RNA using iScript Reverse Transcription Supermix for RT-qPCR (Bio-Rad, Cat. #1708841, USA). Real-Time PCR was performed using the iTaq Universal SYBR Green Supermix (Bio-Rad, Cat. #1725121, USA) on the Bio-Rad CFX Connect Real-Time System using the following primers: P62/SQSTM1 forward primer: 5′- AGGGAACACAGCAAGCT-3′; P62/SQSTM1 reverse primer: 5′-GCCAAAGTGTCCATGTTTCA-3′; GAPDH forward primer: 5′- CCAGAACATCATCCCTGCAT-3′; GAPDH reverse primer: 5′- CAGTGAGCTTCCCGTTCA-3′. Primers for IL-6 and IL-18 were from Bio-Rad PrimePCR™ SYBR® Green Assay. The cycle threshold values were converted to relative gene expression levels using the 2^-ΔΔCt^ method. The data were normalized to that of internal control GAPDH.

### Flow cytometric analysis of autophagosomes (APs)

Autophagic vacuoles, including autophagosomes (APs) and autophagolysosomes (APLs) in cells, were detected using a CytoID Autophagy Detection Kit (Enzo, USA) following the manufacturer’s instruction. The CytoID fluorescent reagents specifically detect the autophagic vacuoles formed during autophagy. Briefly, cells were trypsinized, spun down, and washed twice in phenol red-free DMEM with 2% FBS. The cells were resuspended in 0.5 ml of freshly diluted CytoID reagents and incubated at 37 °C for 30 min. The CytoID fluorescence of cells was immediately analyzed by flow cytometry using a flow cytometer (Attune NxT acoustic focusing cytometer, Life Technologies, USA). The percentage of cells with CytoID staining was used to represent the dynamic balance between AP formation and its degradation via autophagic flux. By combining with an inhibitor of lysosomal degradation (chloroquine, 30 μM) that inhibits autophagic flux, the CytoID assay can be used to determine whether the autophagic vacuole accumulation is due to decrease in autophagic flux.

### Lentiviral transduction

Both control and the mouse sqstm1 shRNA lentil particles were purchased from OriGene (TL515047V). Nfe2l2 shRNA plasmid (TL515053) was also obtained from OriGene. Nrf2 shRNA lenti particles were generated according to the manufacturer’s instructions. *Smpd1*^*−/−*^ SMCs were infected with the lentiviral particle in the presence of polybrene (final concentration was 8 μg/ml). After 48 h transduction, puromycin (1 μg/ml) was added to the media for selection. Surviving cells were allowed to proliferate for another 24 h and were used for downstream analyses. The effect of gene silencing was then analyzed by assessing target mRNA and protein level.

### Immunofluorescence staining

For culture cells, approximately 1 × 10^4^ mouse SMCs were added to the wells of 24-well culture plate containing gelatin-coated coverslips. After being treated with PDGF-BB (30 ng/ml) or the equivalent volume of the vehicle for 24 h, cells were washed with PBS and then fixed with 4% paraformaldehyde for 20 minutes at room temperature. After twice washes with PBS, 0.3% Triton X-100 in PBS was added for 15 min for cell permeabilization. Nonspecific sites were blocked with PBS with 1% BSA and 1% normal donkey serum at room temperature for 1 h. For paraffin-embedded tissue sections, peroxidase treatment in methanol with 0.5% hydrogen peroxide was followed by heat-assisted antigen retrieval in 0.01 M sodium citrate buffer (*p*H 6.0). Blocking was performed for 2 h at room temperature using 2% horse serum followed by overnight incubation with primary antibody at 4 °C. The corresponding secondary antibody was conjugated to Alexa Fluor 488 or Alexa Fluor 647 (Invitrogen). Sections were incubated with secondary antibodies for 1 h at room temperature, followed by cell nucleus staining with DAPI for 10 min. Imaging was performed using Olympus IX73 imaging system.

### Aortic ring sprouting assay

To examine the migration and proliferation of SMCs in an *ex vivo* condition, the mouse aortic ring sprouting assay was performed as described previously^48^. In brief, fresh thoracic aortae were harvested from 12-week old *Smpd1*^*+/+*^ and *Smpd1*^*−/−*^ mice and placed in sterile DMEM buffer. The endothelial layer was removed by the injection of air bubbles. After carefully removing periadventitial fat and connective tissues, the aortae were sliced into ring segments approximately 0.5 mm in thickness. The aortic rings were rinsed with DMEM medium and then embedded in 24-well plates previously coated with 200 µL synthetic basement membrane (Corning^®^ Matrigel^®^) per well on ice. At least 10–15 rings per group were used for each experiment. The plate was placed at room temperature for 15 min and then incubated for 1 h in a humidified incubator (37 °C, 5% CO_2_). Subsequently, 500 μl DMEM culture medium containing 20% FBS was added into each well. The explants were maintained in a humidified incubator (37 °C, 5% CO_2_) with medium replaced every other day. For the treatment group, the culture medium was replaced by fresh DMEM containing 20% FBS and 30 ng/ml PDGF-BB. After 5 days, the outgrowth of cells was observed and imaged using an inverted microscope (Olympus IX73). Digital images of *Smpd1*^*+/+*^ and *Smpd1*^*−/−*^ aortic ring sections were analyzed using Image-Pro Plus software. Each outgrowth emerging directly from the ring was identified (number of sprouts), traced, and branch points marked. ImageJ segmented line tool was then used to measure the longest continuous branch, sum length of all branches (total branch length), and furthest distance from the ring for each outgrowth.

### Statistics analysis

Data are presented as mean ± standard deviation. All in vitro experiments were analyzed by the Student *t*-test or two-way ANOVA with genotype and treatment as category factors, followed by a Bonferroni’s multiple comparisons test if applicable. A Students *t*-test was used to detect significant difference between two groups. The statistical analysis was performed by GraphPad Prism 6.0 software (GraphPad Software, USA). *P* < 0.05 was considered statistically significant.
